# The Association of Cigarette Smoking With Depression and Anxiety: A Systematic Review

**DOI:** 10.1093/ntr/ntw140

**Published:** 2016-05-19

**Authors:** Meg Fluharty, Amy E. Taylor, Meryem Grabski, Marcus R. Munafò

**Affiliations:** 1 ^1^ MRC Integrative Epidemiology Unit (IEU), University of Bristol, Bristol, United Kingdom;; 2 ^2^ UK Centre for Tobacco and Alcohol Studies, School of Experimental Psychology, University of Bristol, Bristol, United Kingdom

## Abstract

**Background:**

Many studies report a positive association between smoking and mental illness. However, the literature remains mixed regarding the direction of this association. We therefore conducted a systematic review evaluating the association of smoking and depression and/or anxiety in longitudinal studies.

**Methods:**

Studies were identified by searching PubMed, Scopus, and Web of Science and were included if they: (1) used human participants, (2) were longitudinal, (3) reported primary data, (4) had smoking as an exposure and depression and/or anxiety as an outcome, or (5) had depression and/or anxiety as the exposure and smoking as an outcome.

**Results:**

Outcomes from 148 studies were categorized into: smoking onset, smoking status, smoking heaviness, tobacco dependence, and smoking trajectory. The results for each category varied substantially, with evidence for positive associations in both directions (smoking to later mental health and mental health to later smoking) as well as null findings. Overall, nearly half the studies reported that baseline depression/anxiety was associated with some type of later smoking behavior, while over a third found evidence that a smoking exposure was associated with later depression/anxiety. However, there were few studies directly supporting a bidirectional model of smoking and anxiety, and very few studies reporting null results.

**Conclusions:**

The literature on the prospective association between smoking and depression and anxiety is inconsistent in terms of the direction of association most strongly supported. This suggests the need for future studies that employ different methodologies, such as Mendelian randomization (MR), which will allow us to draw stronger causal inferences.

**Implications:**

We systematically reviewed longitudinal studies on the association of different aspects of smoking behavior with depression and anxiety. The results varied considerably, with evidence for smoking both associated with subsequent depression and anxiety, and vice versa. Few studies supported a bidirectional relationship, or reported null results, and no clear patterns by gender, ethnicity, clinical status, length to follow-up, or diagnostic test. Suggesting that despite advantages of longitudinal studies, they cannot alone provide strong evidence of causality. Therefore, future studies investigating this association should employ different methods allowing for stronger causal inferences to be made, such as MR.

## Introduction

The high co-occurrence of smoking and mental illness is a major public health concern, and smoking accounts for much of the reduction in life expectancy associated with mental illness.^1^ Many studies report a positive association between smoking and mental illness, with smoking rates increasing with the severity of the disease.^2,3^ Individuals with mental illness also tend to start smoking at an earlier age, smoke more heavily, and are more addicted to cigarettes than the general population. For example, a recent survey suggests that 42% of all cigarettes consumed in England are consumed by those with mental illness, although this includes substance use disorders.^4^ Additionally, while cigarette consumption in the general population has shown a sustained decrease over the past 20 years, consumption among smokers with mental illness has remained relatively unchanged.^1^ There is therefore a pressing need to understand the mechanisms underlying the high rate of smoking in people with mental illness. Here, we focus specifically on the relationship between cigarette smoking and depression and anxiety.

Currently, there are several hypotheses that have been proposed to explain the high rates of smoking in people with depression and anxiety. The self-medication hypothesis postulates that individuals turn to smoking to alleviate their symptoms^5–7^ and therefore suggests that symptoms of depression and anxiety may lead to smoking. An alternative hypothesis is that smoking may lead to depression or anxiety, through effects on an individual’s neurocircuitry that increases susceptibility to environmental stressors. Animal models indicate that prolonged nicotine exposure dysregulates the hypothalamic–pituitary–adrenal system, resulting in hypersecretion of cortisol and alterations in the activity of the associated monoamine neurotransmitter system, whose function is to regulate reactions to stressors,^8^ an effect that appears to normalize after nicotine withdrawal.^9^ The association between smoking and depression/anxiety may also be bidirectional, with occasional smoking initially used to alleviate symptoms, but in fact worsening them over time.^10^ Finally, there may in fact be no causal relationship between smoking and depression/anxiety. Instead, the association may be a product of shared risk factors (eg, common genetic influences)^10,11^ or confounding. Smokers may also report that cigarettes alleviate their symptoms due to the misattribution of withdrawal relief. Given the short half-life of nicotine that results in withdrawal symptoms (including mood symptoms) after a short period of abstinence, smokers may misattribute the relief of short-term withdrawal as reflecting a genuine anxiolytic effect of smoking.^7^ That is, withdrawal symptoms of increased anxiety and negative affect may be misattributed as reflecting genuine mood symptoms, which would lead to the impression that smoking improves mood.

We are therefore presented with multiple different hypotheses regarding whether there is a causal relationship between smoking and depression/anxiety and if so, what the direction of causality underlying this relationship is. While experimental studies are generally not possible, for both practical and ethical reasons, longitudinal studies may help inform our understanding of the causal relationship between smoking and depression/anxiety by clarifying the temporal association. Our study aimed to systematically review the literature comprising longitudinal studies of the associations between smoking and depression/anxiety and conduct meta-analyses where possible. To the best of our knowledge, this is the first systematic review of this literature.

## Methods

### Identification of Studies

We searched PubMed, Scopus, and Web of Science up until August 1, 2015 using the following search terms: depressi*, anxi*, smok*, tobacco, nicotine, cigarette, caus*, cohort, prospective, longitudinal. The term animal* was specified for exclusion. Two authors (MF and AT) reviewed the electronic abstracts, selecting the full-text articles to be included.

### Selection Criteria

Studies were included in the review if they met the following criteria: (1) human participants, (2) smoking as the exposure variable and depression and/or anxiety as the outcome variable, or *vice versa* (depression and/or anxiety as the exposure variable and smoking as the outcome variable), (3) longitudinal study design, and (4) reported primary data not previously reported elsewhere. Studies involving cessation, withdrawal, suicide, or trauma, which recruited participants who were pregnant or diagnosed with a psychiatric illness other than depression or anxiety, or included participants with depression and anxiety comorbid with another psychiatric illness were excluded. Studies not utilizing a validated diagnostic test for depression or anxiety were excluded. Studies investigating the association of parental smoking on offspring outcomes were also excluded, as were all experimental studies (eg, randomized controlled trials of smoking cessation interventions). RCTs as well as secondary analyses of randomized controlled trials were excluded.

### Data Extraction

The following information was extracted from each of the included studies, by one author (MF): type of depression/anxiety (major depression, generalized anxiety disorder, mixed major depression, and generalized anxiety disorder), method of measuring depression/anxiety (self-report via diagnostic test, clinical interview, or physician diagnosis) and scale used (continuous or categorical), smoking behavior (age of smoking onset, smoking status, heaviness of smoking, tobacco dependence, smoking trajectory), sample size, mean age of participants and sex distribution of participants, population sampled (eg, general or clinical), and length of follow up. A 100% data check was performed by the same author (MF) and a 10% data check was independently performed by another author (MG) to identify data extraction errors. Any errors identified were resolved by mutual consent.

### Rationale for not Conducting Meta-analysis

A meta-analysis was not conducted as, even within the general population samples available, there was substantial heterogeneity (age, location, covariates used, time to follow up, and number of times and frequency of outcomes sampled). Additionally, the studies included were not limited to only those examining an a priori hypothesis of mental health and smoking; studies were included if they contained the desired outcome and exposure variables within their data set.

## Results

### Characteristics of Included Studies

**Figure 1.**
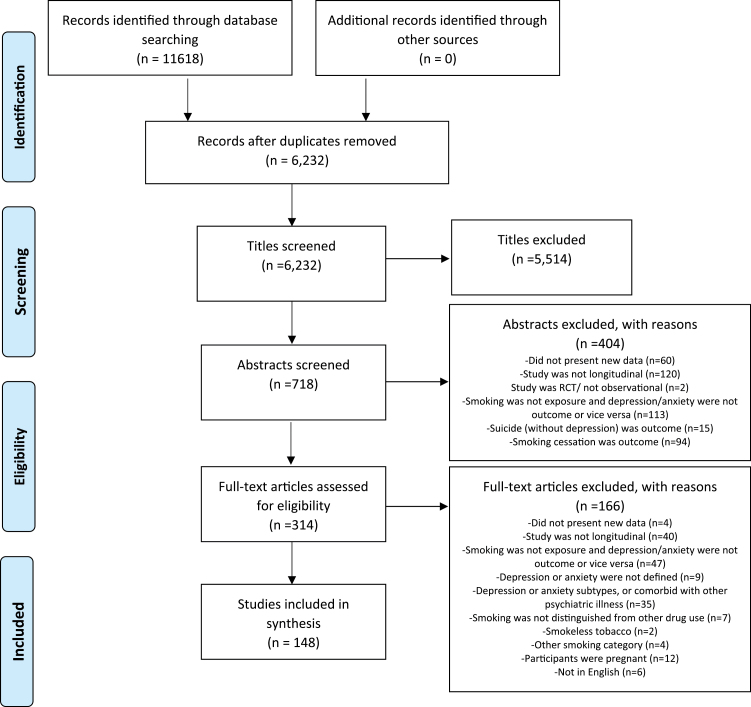
Identification of independent studies for inclusion in systematic review.

Of the 6232 abstracts reviewed, 5514 were excluded on the basis of title and 404 after reviewing the abstract. In total, 314 articles were retrieved and assessed for eligibility, and 148 met inclusion criteria (Figure 1). Details of included studies are provided in Supplementary Table S1 and details of excluded full-text studies in Supplementary Table S2.

Studies ranged in sample size from 59 to 90 627 participants and in length of follow up from 2 months to 36 years. Of the 148 included studies, 99 (67%) recruited male and female participants, 16 (11%) recruited only females and 7 (5%) recruited only males, while 26 (18%) did not report the sex of the participants. In addition, 101 studies (70%) sampled participants from the general population, 15 (10%) from clinical populations, and 16 (10%) from particular ethnic groups, while 16 (10%) had other selection criteria (see Supplementary Table S2).

Unless otherwise stated, the associations described refer to a positive relationship between smoking and depression/anxiety (ie, smoking is associated with increased depression/anxiety, or increased depression/anxiety is associated with increased smoking).

### Smoking Categories

Studies were categorized based on the basis of the smoking behavior(s) they assessed: smoking onset, smoking status, smoking heaviness, tobacco dependence, and smoking trajectory. Studies with measures of daily or weekly cigarette use were included in the smoking heaviness category. Studies that were able to establish the onset of smoking from an initially nonsmoking population were included in the smoking onset category. Studies that measured tobacco dependence, for example, through the *DSM-IV* ^12^ or the Fagerström Test for Nicotine Dependence,^13^ were included in the tobacco dependence category. Studies that tracked the different paths of cigarette smoking uptake and use in a cohort were included in the smoking trajectory category, and studies that defined smokers in purely categorical terms (eg, current, former, and never) were included in the smoking status category. Table 1 summarizes the directions of associations investigated within the studies in each smoking category.

**Table 1.** Directions of Associations Investigated by Smoking Category

Category	Depression			Anxiety			Comorbid depression and anxiety		
	MH into smoking	Smoking into MH	Bidirectional	MH into smoking	Smoking into MH	Bidirectional	MH into smoking	Smoking into MH	Bidirectional
Smoking onset	13	0	1	4	0	2	5	0	1
Smoking status	29	40	8	0	4	1	1	7	0
Smoking heaviness	9	7	2	1	1	0	0	1	0
Tobacco dependence	12	2	1	6	0	0	5	1	0
Smoking trajectory	7	2	0	1	0	0	1	1	0
Any smoking category	70	51	12	12	5	3	12	10	1

**Figure 2.**
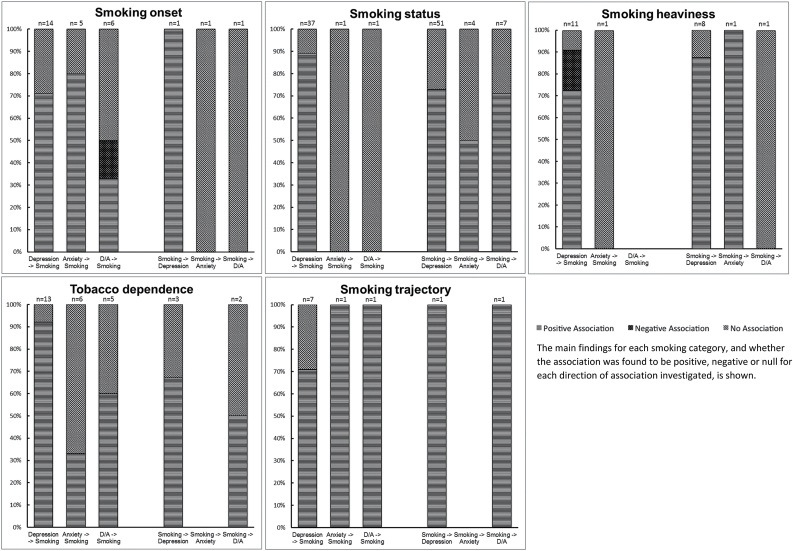
Main outcomes by smoking category.

The number of studies investigating each direction(s) of association for each smoking category is shown. Studies investigating multiple directions are repeated within smoking category. Please note these only include directions investigated and differ from the overall findings within smoking groups detailed in Figure 2. MH = mental health outcome.

### Smoking Onset

A total of 14 studies investigated the association of baseline depression with subsequent smoking onset, of which 10 (71%) found evidence to support this association,^14–23^ while four (29%) found no evidence of an association.^24–27^ Five studies investigated the association of baseline anxiety on smoking onset, of which four (80%) found evidence to support an association with increased risk of smoking onset^24,28–30^ and one (20%) found no evidence of an association.^21^ Six studies investigated the association of comorbid depression and anxiety with later smoking onset, of which two (33%) found evidence to support this association,^31,32^ while one (17%) reported comorbid depression and anxiety was associated with reduced risk of smoking onset^33^ and three (50%) found no evidence of an association.^34–36^ One study investigated the association of smoking onset with later depression, finding evidence for this association.^15^ One study investigated the association of smoking onset with later anxiety, finding no evidence for this association.^21^ Additionally one study investigated the association of smoking onset with later comorbid depression and anxiety, finding no evidence for this association.^31^ These findings are summarized in Figure 2.

### Smoking Status

A total of 37 studies investigated the association of baseline depression with subsequent smoking status, of which 33 (89%) found evidence to support this association,^21,37–66^ while four (11%) found no evidence of an association.^67–70^ One study investigated the association of anxiety with later smoking status, finding evidence of an association.^28^ One study investigated the association of comorbid depression and anxiety with later smoking status, finding no evidence of an association.^71^

A total of 51 studies investigated the association of smoking status with later depression, of which 37 (73%) found evidence to support this association,^21,25,47,57,65,70,72–102^ while 14 (27%) found no evidence of this association.^28,38,48,64,69,103–111^ Four studies investigated the association of smoking status with later anxiety, of which two (50%) found evidence to support this association,^28,112^ while two (50%) found no evidence of an association.^21,103^ Seven studies investigated the association of smoking status with later comorbid depression and anxiety, of which five (71%) found evidence to support this association,^35,113–116^ while two (29%) found no evidence of an association.^117,118^ These findings are summarized in Figure 2.

### Smoking Heaviness

A total of 11 studies investigated the association of baseline depression with subsequent heaviness of smoking, of which eight (73%) found evidence that depression was associated with heavier rates of smoking,^22,119–125^ while two (18%) found that depression was associated with reduced heaviness of smoking^26,126^ and one (09%) found no evidence of an association.^127^ One study investigated the association of baseline anxiety with subsequent smoking heaviness and found no evidence of an association.^124^ Eight studies investigated the association of heaviness of smoking with later depression, of which seven (88%) found evidence to support this association,^11,82,95,102,125,127,128^ while one (13%) found no evidence of an association.^129^ One study investigated the association of heaviness of smoking with later anxiety and found evidence to support this association.^130^ One study investigated the association of heaviness of smoking with later comorbid depression and anxiety, finding no evidence of an association.^117^ These findings are summarized in Figure 2.

### Tobacco Dependence

A total of 13 studies investigated the association of baseline depression with subsequent tobacco dependence, of which 12 (92%) found evidence to support this association^29,120,131–140^ while one (8%) found no evidence of an association.^141^ Six studies investigated the association of baseline anxiety with later tobacco dependence, of which two (33%) found evidence to support this association,^140,142^ while four (67%) found no evidence of an association.^132,137,139,143^ Five studies investigated baseline comorbid depression and anxiety with subsequent tobacco dependence, of which three (60%) found evidence to support this association, ^144–146^ while two (40%) found no evidence of an association.^35,147^ Three studies investigated the association of tobacco dependence with later depression, of which two (67%) found evidence to support this association,^6,132^ while one (33%) found no evidence of an association.^148^ Two studies investigated the association of tobacco dependence with later comorbid depression and anxiety, of which one (50%) found evidence to support this association,^149^ while one (50%) found no evidence of an association.^147^ These findings are summarized in Figure 2.

### Smoking Trajectory

A total of seven studies investigated the association of baseline depression with smoking trajectory, of which one (14%) reported that depressive symptoms were associated with accelerated cigarette use,^150^ three (43%) reported that depressive symptoms were associated with early smoking onset,^17,43,151^ one reported that depressive symptoms were associated with late onset smoking^152^ and two (29%) found no evidence of an association.^153,154^. One study reported evidence of an association of baseline anxiety with early and late onset smoking patterns.^155^ Another study reported evidence of an association of baseline comorbid depression and anxiety with late onset smoking as opposed to experimental smoking.^156^ One study reported that individuals in (smoking) starter and maintaining groups were more likely to be depressed at follow up compared with nonsmoking groups.^157^ Finally, one study reported evidence that early onset smokers developed depression and anxiety approximately five years earlier than late onset smokers.^158^ These findings are summarized in Figure 2.

### Bidirectional Studies

Sixteen (11%) of the 148 included studies investigated the association between smoking behavior and mental health bidirectionally (ie, both the association between baseline mental health and later smoking behavior and baseline smoking behavior and later mental health). Of these, seven (44%) reported evidence in support of a bidirectional relationship between depression and smoking^15,21,47,57,65,125,132^ and one (9%) reported evidence in support of a bidirectional relationship between anxiety and smoking.^28^

### Sex Differences

A total of eight studies (7% of all studies including both males and females) reported that the relationship between smoking and depression/anxiety differed between males and females. Two studies reported that depression was associated with subsequent smoking behavior only in males,^23,64^ while one study reported depression was associated with subsequent smoking only in females^66^ and one study reported that anxiety was associated with later smoking behavior only in females.^140^ Additionally, one study reported evidence that smoking status in men was associated with later depression,^101^ and two studies reported evidence that smoking status had a stronger association with later depression in females than males.^97,157^ Finally, one study reported a bidirectional relationship between smoking and depression that was only observed in females.^57^

### Clinical Studies

Five studies investigated participants with cardiovascular problems. One study reported evidence that depression was associated with subsequent smoking behavior.^44^ The other four reported that smoking status was associated with later depression.^80,83,87,88^ Other studies of clinical populations generally reported evidence of an association between smoking and the onset of depression.

### Ethnic Differences

Five studies recruited participants of East Asian descent (China, Japan, and South Korea), with two studies reporting evidence that depression was associated with later smoking behavior ^41,48^ and one study reporting no evidence of an association.^70^ Additionally, two studies reported evidence for an association between smoking status and later depression,^70,99^ while two studies reported no evidence that smoking status was associated with subsequent depression.^48,108^ Three studies recruited African American participants, with two studies reporting evidence that depression was associated with later smoking behavior,^54,64^ one study reporting no evidence that depression was associated with subsequent smoking onset,^153^ and one study reporting no evidence that smoking was associated with the onset of depression.^64^ Four studies recruited both African American and Hispanic participants, with three studies reporting that depression and anxiety were associated with subsequent smoking trajectories,^43,131,156^ while one study reported that smoking heaviness was associated with the onset of anxiety.^130^ Other studies of specific ethnic groups generally reported evidence of an association between smoking and later depression and anxiety.

### Additional Analyses

No clear pattern of results was apparent when studies with different lengths of follow up were considered separately (see Supplementary Table S3). Additionally, the findings did not vary substantially between studies using different tests (interview vs. self-diagnostic test) or scales (continuous vs. categorical) to diagnose depression or anxiety (see Supplementary Table S4).

## Discussion

In general, the findings across the studies in our systematic review were inconsistent. Nearly half of the studies reported that baseline depression or anxiety was associated with some type of later smoking behavior, whether it be the onset of smoking itself, increased smoking heaviness, or the transition from daily smoking into dependence. These findings support a self-medication model, suggesting that individuals smoke to alleviate psychiatric symptoms.^5,6^ However, over a third of the studies found evidence for a relationship in the opposite direction whereby smoking exposure at baseline was associated with later depression or anxiety, supporting the alternative hypothesis that prolonged smoking increases susceptibility to depression and anxiety.^8,9^ Of course, these two putative causal pathways are not mutually exclusive, but interestingly there were relatively few studies reporting evidence for a bidirectional model relationship between smoking and depression and anxiety. One possible reason for this is that many studies only measured or analyzed the variables in the direction of their a priori hypothesis. For example, studies examining factors for depression in later life measured smoking as a possible factor but typically did not analyze the association of baseline depression with later smoking. Moreover, few studies reported null results; often these were only included alongside positive results relating to another outcome. Additionally, it is possible the associations observed between smoking and mental health are a result of shared genetic and environmental factors.^6^

There are a number of limitations that should be considered when interpreting these results. First, the studies included in this review varied substantially in population sampled, with some recruiting from the general population and others selectively recruiting by sex, ethnicity, clinical population, or some other characteristic (eg, at-risk adolescents). This introduced substantial heterogeneity into the review, thus making meta-analysis inappropriate. The substantial heterogeneity between study populations could be responsible for the inconsistent results observed, and future reviews should consider analyzing different populations individually. Second, there was also substantial variation in study designs, including the length of follow up (between 2 months and 36 years) and confounders adjusted for. Measurement of depression or anxiety was based on a wide range of different diagnostic tests, with different cutoffs for determining clinical status. Sample size also varied substantially between studies, ranging from 59 to 90 627, suggesting that some smaller studies may be inadequately powered. This may lead to an increased likelihood of false positives since, among statistically significant findings, power declines the ratio of true positives to false positives decreases.^159^ This is because while 5% of null associations will be falsely declared as significant (assuming a 5% alpha level), the number of true positives correctly identified will decline as power declines (eg, from 80% of true associations correctly declared as significant in high powered studies to, say, only 20% in low powered studies).^159^ However, it is also worth noting that very large samples may detect statistically significant associations that are unlikely to be of clinical or population health importance.

Third, we only included published studies, and while the inclusion of unpublished studies may increase the likelihood of including lower quality work that has not been peer reviewed, it may also decrease publication bias, in which studies are only published if they have positive results. By expanding our search to include non-published studies, it is possible we may have found more instances of null results. Fourth, we did not investigate whether quality of the individual studies was related to the nature of the results reported. However, this would be challenging, given the diversity of study designs among the included studies. Fifth, while we were able to categorize and investigate a range of different smoking behaviors, the same level of detail was not available for depression and anxiety. Future reviews should investigate individual symptomology (eg, negative affect, somatic features, etc.) and their relationship with smoking behavior, as previous research has indicated that specific symptoms may be differentially associated with smoking motivations and tobacco withdrawal.^160–162^ However, this analysis was not possible with the data reviewed here. Sixth, we only focused on depression, anxiety, or comorbid depression and anxiety. However, several studies identified during screening included depression or anxiety subtypes (eg, post-traumatic stress disorder or social anxiety). These were excluded in order to maximize comparability among included studies. Future studies should explore whether there is a more consistent pattern of relationship between smoking behavior and other diagnostic categories. However, given the disparate results, we observed in our more focused review, it is perhaps unlikely that clear relationships will emerge.

Despite the advantages of longitudinal studies, they cannot by themselves provide strong evidence of causality. However, applying latent variable mixture modeling to establish group-based trajectories, as some studies identified did, may help to identify different patterns within the data that may have otherwise gone unnoticed. Rather than clustering individuals into simply “smokers” and “nonsmokers,” mixture modeling can identify various groups such as “experimenters,” “early onset,” “late onset,” “stable,” or “late escalating” smokers.^163^ This approach could provide insight into the type or critical age of smokers vulnerable to mental illness, or vice versa. It’s likely that our review did not yield more of these studies, as we did not include “trajectory” in our search terms. Future reviews should include an exhaustive search, including a variety of terms such as mixture modeling, latent class analysis, and latent trajectory analysis in addition to the term trajectory.

Additionally, future studies should therefore employ methods that enable stronger causal inference, such as Mendelian randomization (MR).^164^ This approach uses genetic polymorphisms that have been previously shown to be robustly associated with one of the exposures of interest; for example, the *CHRNA5-A3-B4* gene cluster is associated with smoking quantity and tobacco dependence^165,166^ and has been used in a number of MR studies.^160^ It is based on the principle that an individual inherits a random assortment of genes from their parents, and these genes should not be associated with potential confounders.^10^ Therefore, in theory, a robust genetic influence to a particular exposure (eg, smoking) would be comparable to a randomized trial in which individuals are assigned to a high- or low-exposure group.^164^ In addition, environmental factors cannot affect the genes that an individual is born with, so analyses are not subject to reverse causality or residual confounding. Two studies that have used MR have found no evidence to support a causal association between smoking and depression and anxiety,^117,167^ while another found evidence to suggest that smoking was associated with lower odds of depression during pregnancy.^168^ The results of these studies suggest that observational findings of an association of smoking status with later psychological distress may be a result of shared vulnerability, residual confounding, or reverse causality (eg, psychological distress associated with later smoking behavior).^167^ However, this review yielded the most findings in the direction of psychological distress associated with later smoking behavior. This review found slightly more evidence to support a direction of psychological distress predicting later smoking behavior, which is not inconsistent with these MR studies.^167,168^ However, while both depression and anxiety are highly heritable,^169,170^ genomewide association studies have not identified genetic variants robustly and strongly associated with these outcomes.^171^ Therefore, it is not currently possible to use MR to examine whether depression and anxiety are associated with smoking behavior, although this is likely to change in the near future as larger genomewide association studies of depression and anxiety emerge. Until such genomewide association studies emerge, it is not possible to directly test the causal hypothesis in this direction.

In summary, we found overall inconsistent findings regarding whether smoking leads to depression and anxiety, depression and anxiety results in smoking or increased smoking behavior, or there is a bidirectional relationship between the two. This conflicting evidence suggests the need for future studies to focus on different methodologies, such as MR, which will allow us to draw stronger causal inferences.

## Supplementary Material

Supplementary Tables S1 to S4 can be found online at http://www.ntr.oxfordjournals.org

## Funding

This work was supported by a University of Bristol postgraduate research scholarship to MF, and the Medical Research Council (MC_UU_12013/6).

## Declaration of Interests

*None declared.*

## Supplementary Material

Supplementary Data
