# Supplementary material for: The Association of Cigarette Smoking With Depression and Anxiety: A Systematic Review
Source: Nicotine Tob Res. 2016 May 19;19(1):3–13. doi: 10.1093/ntr/ntw140 (PMC5157710; doi:10.1093/ntr/ntw140)
Supplement: Supplementary Data [file supp_19_1_3__index.html]

The Association of Cigarette Smoking with Depression and Anxiety: A systematic review — The Association of Cigarette Smoking With Depression and Anxiety: A Systematic Review — The Association of Cigarette Smoking With Depression and Anxiety: A Systematic Review — Supplementary Data 

# The Association of Cigarette Smoking With Depression and Anxiety: A Systematic Review

## Supplementary Data

Data files

- Supplementary Data - Supplementary Data
- Supplementary Data - Supplementary Data
- Supplementary Data - Supplementary Data
