# Supplementary material for: The Association of Cigarette Smoking With Depression and Anxiety: A Systematic Review
Source: Nicotine Tob Res. 2016 May 19;19(1):3–13. doi: 10.1093/ntr/ntw140 (PMC5157710; doi:10.1093/ntr/ntw140)
Supplement: Supplementary Data [file supp_ntw140_Fluharty_Smoking_Depression_Supplementary_Tables_S3_S4.docx]

**Supplementary Table S3: Main findings stratified by length to follow-up**

| **Direction of association** | **Finding** | **≤ 1 years** | **> 1 to 5 years** | **6 to 10 years** | **> 10 years** |
| --- | --- | --- | --- | --- | --- |
| **Depression/anxiety exposure into smoking outcome** | **Evidence for** | 16 (48%) | 28 (42%) | 11 (35%) | 14 (29%) |
|  | **Evidence against** | 3 (9%) | 14 (21%) | 1 (3%) | 11 (23%) |
| **Smoking exposure into depression/anxiety outcome** | **Evidence for** | 7 (20%) | 13 (20%) | 14 (45%) | 17 (35%) |
|  | **Evidence against** | 5 (14%) | 8 (12%) | 3 (10%) | 6 (13%) |
| **Bidirectional smoking and mental health outcome** | **Evidence for** | 4 (11%) | 3 (46%) | 2 (6%) | 0 (0%) |
|  | **Evidence against** | - | - | - | - |

The main findings of each study stratified by length to follow-up are summarised.

**Supplementary Table S4: Main findings stratified by type of diagnostic test used**

| **Direction of association** | **Finding** | **Interview*** | **Diagnostic test** | **Continuous** | **Categorical** |
| --- | --- | --- | --- | --- | --- |
| **Depression/anxiety exposure into smoking outcome** | **Evidence for** | 20 (33%) | 51 (43%) | 38 (41%) | 29 (32%) |
|  | **Evidence against** | 11 (18%) | 16 (14%) | 12 (13%) | 17 (19%) |
| **Smoking exposure into depression/anxiety outcome** | **Evidence for** | 18 (30%) | 32 (27%) | 25 (27%) | 29 (32%) |
|  | **Evidence against** | 8 (13%) | 13 (11%) | 11 (12%) | 12 (13%) |
| **Bidirectional smoking and mental health outcome** | **Evidence for** | 3 (5%) | 6 (5%) | 6 (7%) | 3 (3%) |
|  | **Evidence against** | - | - | - | - |

The main findings of each study stratified by type of diagnostic test and scale used to define depression or anxiety are summarised.

*Includes two studies that used physician diagnosis.
